# Sensors for Robots

**DOI:** 10.3390/s24061854

**Published:** 2024-03-14

**Authors:** Xin Zhao, Mingzhu Sun, Qili Zhao

**Affiliations:** 1 National Key Laboratory of Intelligent Tracking and Forecasting for Infectious Diseases, The Engineering Research Center of Trusted Behavior Intelligence, Ministry of Education, The Tianjin Key Laboratory of Intelligent Robotics (tjKLIR), Institute of Robotics and Automatic Information System (IRAIS), Nankai University, Tianjin 300350, China; sunmz@nankai.edu.cn (M.S.); zhaoqili@nankai.edu.cn (Q.Z.); 2 The Institute of Intelligence Technology and Robotic Systems, Shenzhen Research Institute of Nankai University, Shenzhen 518083, China

## 1. Introduction

Currently, robots are playing significant roles in industry [1], life sciences [2], education [3], medicine [4], social services [5], and the military [6]. Robots will undoubtedly play increasingly important roles in the future life of human beings [7]. Sensors are key components of robots, enabling them to perceive additional environmental information, just like the scanners of the famous R2D2 Robot in Star Wars movies [8]. They are the basis of the self-adaptive abilities and automation control abilities of robots. Therefore, novel sensing techniques and advanced sensor applications for robots have received increasing attention worldwide [9].

Novel sensor technologies for robotics have been developed to detect new environmental parameters or improve the measurement accuracy of the present parameters [10]. To achieve this, novel design [11], fabrication [12], and calibration methods [13] have been invented to improve the detection sensitivity of the sensors. Besides the aforementioned sensor innovations, advancements in the integration [14], diffusion [15], and processing methods [16] of the detected signals have been made to improve the SNR and acquire useful information from gross signals. Based on these new technologies, novel force [17], vision [18], tactile [11], and auditory [19] sensors have been reported in recent years.

With the rapid development of swarm robots and multi-agent systems, multi-sensors [20], reconfigurable sensors [21], and cyber–physical sensing systems [22] have been developed to detect the parameters of each robot or agent. Because each body of the above system is usually far from the other, wireless sensor networks [23], multi-sensing intelligent systems [20], and sensor systems for human–robot interactions [24] have been developed to facilitate the communication and processing of the signals of each robotic body.

With the widespread application of robots, sensors for novel robotic applications have made significant progress. For example, vision sensors and vision algorithms for motive object tracking and robot navigation have been widely applied in mobile robots [25], unmanned aerial vehicles [26], and underwater vehicles [27]. In addition to the applications in traditional research areas, robot sensing technologies have also been widely applied in interdisciplinary areas, including life science [2], material sciences [28], physical sciences [29], and micro-nano manipulations [30].

## 2. Overview of Contribution

In this context, this topical collection includes ten papers focusing on the latest advancements in the field of sensors for robots and robotic sensing applications. Each of the ten original contributions accepted for publication in this Special Issue has undergone a rigorous review process involving a minimum of two expert reviewers across at least two rounds of revision. The studies featured in the current topical collection are summarized as follows.

In Contribution 1, the authors proposed a multi-path scattering model for virtual vegetation. On this basis, a passive microwave imaging algorithm was introduced to reconstruct vegetation images based on the scattering data from the multi-path model received by the UAV (Unmanned Aerial Vehicle) robot. The combination of the electromagnetic scattering model and image processing method contributes to understanding the image results and the multi-path scattering mechanisms of vegetation, which provide a reference for the research and development of microwave imaging systems of UAV robot networking using satellite transmitting signals.

In Contribution 2, the authors proposed a novel reinforcement learning-based hierarchical control framework to enable a snake robot with an onboard camera to realize autonomous self-localization and path following. A series of simulations and hardware experimental results validate that the proposed method is capable of achieving precise and fast convergence for the path-following tasks for a snake robot with autonomous visual perception.

Contribution 3 by Yang et al. discussed the coverage of practical models with varying intensities. This study analyzed the properties of the effective coverage of multi-node teams consisting of a given number of nodes. Numerical analyses and simulations for 3-node and n-node teams (n ≥ 4) were conducted separately. For the 3-node cases, the relations between the side lengths of equilateral triangle formation and the effective coverage of the team equipped with two different types of models were inspected. For the n-node cases (n ≥ 4), the effective coverage of a team in three formations, namely regular polygon, regular star, and equilateral triangular tessellation (for n = 6), were investigated. This study provides useful insights and guides for the development of practical devices for coverage applications.

In Contribution 4 by Imtiaz et al., the target was to have the agent learn to perform prehensile and non-prehensile robotic manipulations to improve the efficiency and throughput of the pick-and-place task. To achieve this target, this study specified the problem as a Markov decision process (MDP) and deployed a deep reinforcement learning (RL) temporal difference model-free algorithm known as the deep Q-network (DQN). Experiments carried out under a range of complex scenario test cases show promising results compared to a baseline deep learning approach and a ResNet architecture-based approach.

In Contribution 5, Shi et al. presented a design and a control algorithm for a robot used for grinding the surfaces of large, curved workpieces with unknown parameters, such as wind turbine blades. First, the structure and motion mode of the grinding robot were determined. Considering that fuzzy PID has the advantages of variable parameters and strong adaptability, this study proposed a force/position hybrid control strategy based on fuzzy PID. The experimental results of robotic grinding verified the feasibility and effectiveness of the proposed control strategy compared with the artificial results.

Contribution 6 by Li et al. proposed a robotic intracellular pressure measurement method using a traditional micropipette electrode system setup, in which intracellular pressure, a key physical parameter of the intracellular environment, has been found to regulate multiple cell physiological activities and impact cell micromanipulation results. This measurement method does not necessitate specialized and expensive equipment and causes limited damage to cell viability. The experimental intracellular pressure measurement value for porcine oocytes, which is consistent with the value reported in the relevant literature, verifies the effectiveness of the proposed measurement method. Moreover, a 90% survival rate of operated oocytes was obtained after measurement, indicating limited damage to cell viability.

In Contribution 7, Isabel et al. presented a novel active device for shoulder rotation based on force control. This device is designed for shoulder rehabilitation and can perform three different exercises. This device uses a force-control architecture and an adaptive-speed PI controller. The study conducted in 12 shoulder rehabilitation sessions showed a significant improvement in shoulder rotation. The patients reported high levels of device acceptance. This device could be a valuable tool for shoulder rehabilitation and improve the efficiency of physiotherapy services.

In Contribution 8, Shu et al. proposed a method for the accurate 3D posture sensing of soft actuators. It utilizes miniaturized sponge-resistive materials and LSTM neural networks. This method considers the hysteresis and non-linear sensing signals. It was demonstrated on a 3-DOF pneumatic bellow-shaped actuator with nine sensors. The sensors exhibited a low error rate in predicting the position of the actuator tip.

In Contribution 9, Rostkowska et al. focused on appearance-based localization using catadioptric cameras. The authors proposed an efficient neural network architecture for recognizing indoor scenes from distorted images. The system was optimized for real-time inference on edge devices. Transfer learning and fine-tuning of a limited number of catadioptric images yielded the best results. The system was tested on small-scale datasets and compared with state-of-the-art systems. The proposed system performed well in terms of accuracy and inference time, providing a cost- and energy-efficient solution for appearance-based localization.

In contribution 10, Weidenbach et al. discussed the challenges of estimating the 6D poses of transparent objects for robotic manipulation. Robotic manipulation requires accurate object pose knowledge. Estimating the poses of transparent objects is challenging because of unreliable depth data and the different effects of clutter. The authors proposed a transparency-aware multi-segmentation approach and transparency-aware bounding boxes. The proposed approach improves pose estimation for transparent objects without modifying the pose estimator. The study demonstrated a 4.3% increase in the ADD-S AUC value using transparency-aware segmentation. The transparency-aware segmentation can be applied to any dataset that provides a 3D model of an object and its ground-truth pose.

## 3. Conclusions

In summary, this topical collection includes ten novel and interesting works in the area of sensors for robots and robotic sensing applications. We would like to express our deepest gratitude to the Managing Team of *Sensors* for their continuous support throughout the preparation of this collection. We greatly appreciate all the contributing authors and the anonymous expert reviewers for their invaluable efforts in selecting submissions of the utmost quality.
